# Case Report: A New Peroxisome Proliferator-Activated Receptor Gamma Mutation Causes Familial Partial Lipodystrophy Type 3 in a Chinese Patient

**DOI:** 10.3389/fendo.2022.830708

**Published:** 2022-03-29

**Authors:** Xi Chen, Zhiqiang Ma, Peng Chen, Xiuli Song, Weihua Li, Xuefeng Yu, Junhui Xie

**Affiliations:** ^1^Division of Endocrinology, Department of Internal Medicine, Tongji Hospital, Tongji Medical College, Huazhong University of Science and Technology, Wuhan, China; ^2^Department of Internal Medicine, Branch of National Clinical Research Center for Metabolic Disease, Wuhan, China; ^3^Division of Cardiology, Departments of Internal Medicine, Liyuan Hospital, Tongji Medical College, Huazhong University of Science and Technology, Wuhan, China; ^4^Division of Cardiology, Departments of Internal Medicine and Genetic Diagnosis Center, Tongji Hospital, Tongji Medical College, Huazhong University of Science and Technology, Wuhan, China; ^5^Department of Internal Medicine, Hubei Key Laboratory of Genetics and Molecular Mechanism of Cardiological Disorders, Wuhan, China

**Keywords:** lipodystrophy, peroxisome proliferator-activated receptor gamma, adipose tissue, insulin resistance, diabetes mellitus, case report

## Abstract

**Purpose:**

Familial partial lipodystrophy type 3 (FPLD3) is an autosomal dominant disease. Patients typically present with loss of adipose tissue and metabolic complications. Here, we reported a Chinese FPLD3 patient with a novel *PPARG* gene mutation.

**Methods:**

A 16-year-old female patient and her relatives were assessed by detailed clinical and biochemical examinations. Sequencing was performed by using the extracted DNA. Moreover, we identified FPLD3 patients from previous studies, and according to the protein region affected by the gene mutation. We divided the patients into the DNA-binding domain (DBD) group or the ligand-binding domain (LBD) group, and compared the clinical features between the two groups.

**Results:**

We identified a novel gene mutation affecting the LBD of PPARγ c.929T > C (p.F310S). This mutation leads to the substitution of a phenylalanine by a serine. In our case, subcutaneous fat was significantly diminished in her face, hips and limbs. The patient was also presented with insulin resistance, diabetes mellitus, hypertriglyceridemia, fatty liver, liver dysfunction, albuminuria and diabetic peripheral neuropathy. After literature review, a total of 58 FPLD3 patients were identified and we found no difference in clinical features between the DBD group and LBD group (all P > 0.05).

**Conclusions:**

A Chinese FPLD3 patient with a novel *PPARG* gene mutation is described. Our case emphasized the importance of physical examination and genetic testing in young patients with severe metabolic syndromes.

## Introduction

Familial partial lipodystrophy (FPLD) is a rare autosomal dominant disease characterized by lipoatrophy of the extremities (1). The disease is accompanied by metabolic abnormalities including insulin resistance (IR), diabetes mellitus (DM), and dyslipidemia (2, 3). According to different pathogenic genes, six types of FPLD and several other forms have been described.1 Mutations in these genes may affect adipocyte differentiation, cell membrane integrity, DNA repair, lipid droplet formation and lipolysis. FPLD type 3 (FPLD3) is induced by mutations in the *PPARG* gene, encoding for peroxisome proliferator-activated receptor gamma (PPARγ) (4). PPARγ, which belongs to the family of nuclear receptors, is required for adipocyte differentiation and regulation of insulin sensitivity (5). *PPARG* mutations may inhibit adipocyte differentiation and consequently cause lipotoxicity, low-grade inflammation, altered adipokine secretion and ectopic fat tissue accumulation. To date, more than 60 patients with FPLD3 have been reported.

Similar to other nuclear receptors, PPARγ contains an N-terminal domain (A/B), a DNA-binding domain (DBD), a ligand-binding domain (LBD), and a hinge region (D) that separates the LBD from the DBD (6). Until now, most mutations causing FPLD3 have been located on the DBD or LBD. Nevertheless, the link between mutations located on the DBD or the LBD and clinical phenotypes is currently unclear.

Here, we report the first case of FPLD3 with a novel *de novo PPARG* mutation. In addition, we collected phenotypic data for FPLD3 from previous literature between 1999 and 2021, and explored the correlations between mutations located in different domains and clinical phenotypes.

## Materials and Methods

### Patient and Family

We examined four members of a Chinese family, with one affected patient. Informed consent was obtained from all the participants for clinical and molecular genetic studies, after a full explanation of the purpose and nature of all procedures used. Blood samples were taken and analyzed by standard laboratory methods at the Institute of Laboratory Medicine, Tongji Hospital, Tongji Medical College, Huazhong University of Science and Technology, Wuhan, China.

### DNA Analysis

The genomic DNA of the patient and his parents was extracted from the whole blood using a DNeasy Blood and Tissue Kit (Qiagen) according to the manufacturer’s protocol. Different barcode-ligated libraries were individually constructed using genomic DNA and were combined at equal molar ratios for sequencing. The libraries were sequenced on an Illumina platform (HiSeq2500; Illumina, Inc.).

To identify novel potential pathogenic variants, we first removed benign variants in the ClinVar database (https://www.ncbi.nlm.nih.gov/clinvar/) and single nucleotide polymorphisms (SNPs) (MAF > 0.1%) from the Exome Aggregation Consortium (ExAC) database (http://exac.broadinstitute.org/). Furthermore, noncoding variants (up/downstream, intron) and synonymous variants were also removed. The novel missense variants were evaluated by scoring with Polyphen-2 (http://genetics.bwh.harvard.edu/pph2). Finally, the pathogenic variant was identified following the American College of Medical Genetics and Genomics (ACMG) standards and guidelines for the interpretation of sequence variants.

To validate the identified potential pathogenic variants, we performed Sanger sequencing. The PCR amplifications were performed using individually designed primers. Amplification products were sequenced using the IDT capture kit on an Illumina platform.

### Structure Analysis

Protein structural analysis was performed employing the crystal structure of human PPARγ isoform 2. The protein sequences were retrieved from the NCBI Protein Sequence Database (http://www.ncbi.nlm.nih.gov/protein). The wild-type and mutant models were built using Swiss-Model (http://swissmodel.expasy.org/). Structural analysis of both models and presentation of the mutated amino acids was performed with Pymol (The PyMOL Molecular Graphics System, Version 2.0 Schrödinger).

### Data Collection and Analysis

We retrieved relevant published literature from PubMed from Jan 1999 to June 2021. Phenotypic data for patients with FPLD3 were collected and divided into two groups (the DBD group and the LBD group) according to the region of the protein domain in which the mutation was located. The inclusion criteria were as follows: i) all patients met the defined core clinical characteristics for the detection of FPLD from the AACE consensus statement published in 2013 (7), ii) patients were subjected to genetic testing to identify pathogenic mutations in *PPARG*, iii) pathogenic mutations were located on the DBD or LBD, and iv) detailed medical history and patient characteristics were described. Cases with frame shift mutation or nonsense mutation were excluded. For categorical data, we used a chi-square or Fisher’s exact test. A significant difference was set at a p value < 0.05. The software used was SPSS 15.0.

## Results

### Case Presentation

A 16-year-old female was admitted to our hospital in September 2020 with hyperglycemia. The patient presented typical symptoms of DM, such as polyphagia, polydipsia, and polyuria, over the last 7 months. In May 2020, she was initially diagnosed with type 2 DM (T2DM) and medicated with 1500 mg/day metformin and 60 IU/day insulin (insulin aspart injection 10 IU at breakfast, 10 IU at lunch and 10 IU at dinner, insulin degludec 30 IU at bedtime) at a local hospital. However, the patient’s glycemic control was poor, with a fasting gluco8se range (10-14 mmol/L) and postprandial glycemia range (12-18 mmol/L). In addition, she had a previous history of atrial septal defect repair in 2009 and a 7-year history of adenotonsillar hypertrophy. There was no family history of DM.

**Figure 1:**
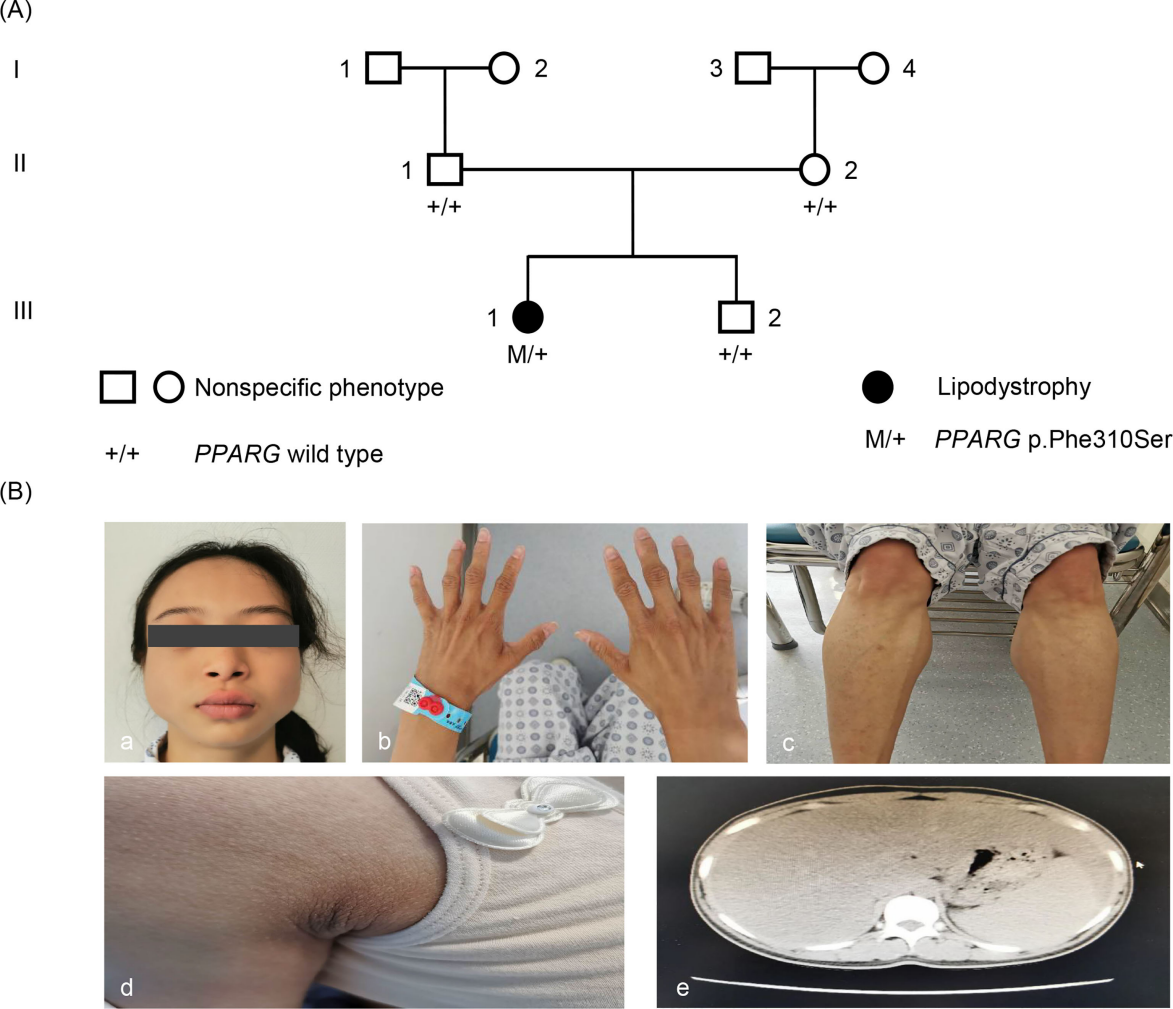
Pedigree and phenotype. **(A)** Pedigree chart: The patient is indicated by dark symbols. The patient carrying the heterozygote mutation c.929T *>* C is marked by M/+. Tested family members without the mutation are marked by *+/+*. **(B)** Phenotype: a, c, and d, the patient presented with loss of subcutaneous fat, especially in the face, hands, and lower limbs. b, Patient had acanthosis nigricans in axillaris. e, Abdominal CT showed liver and spleen enlargement and fatty liver.

Physical examination showed normal blood pressure. Her BMI was 17.5 kg/m^2^ (height, 155 cm; weight, 42 kg). The patient’s waist circumference was 74 cm. Subcutaneous fat was significantly diminished in her face, hips and limbs, with prominent gastrocnemius (**Figure 1**). There was no increase in back and abdominal fat. She had phlebectasia in the lower legs and acanthosis nigricans in the axillaris (**Figure 1**). The inferior margin of the liver was palpable at the level of the umbilicus. The patient had normal menstrual cycles, and no hirsutism.

Laboratory test findings on admission were as follows: her glycated hemoglobin was 8.4% (reference range (RR): 4%–6%), alanine aminotransferase was 54 U/L (RR: < 35 U/L), aspartate aminotransferase was 84 U/L (RR: < 40 U/L), triglycerides were 6.17 mmol/L (RR: 0.6–1.84 mmol/L), total cholesterol was 3.7 mmol/L (RR: < 0.51 mmol/L), high-density lipoprotein was 0.75 mmol/L (RR: > 1.5 mmol/L), low-density lipoprotein was 1.36 mmol/L (RR: < 3.37 mmol/L), total bilirubin was 33.8 μmol/L (RR: 1.71–21 μmol/L), direct bilirubin was 10.3 μmol/L (RR: < 7.32 μmol/L), indirect bilirubin was 23.5 μmol/L (RR: < 14 μmol/L), uric acid was 412.0 μmol/L (RR: < 357 μmol/L), 24-hour urinary microalbumin was 238.4 mg/24 h (RR: < 150 mg/24 h), and thyroid-stimulating hormone was 5.46 μIU/ml (RR: 0.4–2.5 μIU/ml). An oral glucose tolerance test (OGTT) was performed and showed IR (fasting glucose, insulin and C-peptide were 14.25 mmol/L, 22.68 μU/mL, and 3.7 μg/L, respectively, which increased to 17.00 mmol/L, 130.60 μU/mL and 11.06 μg/L at 120 min after a 75 g oral glucose load, respectively). Other indicators, including cortisol, growth hormone, estrogen, androgen, progesterone, luteinizing hormone, follicle-stimulating hormone, prolactin, pancreatic damage and insulin-like growth factor-1, were normal. Abdominal CT showed liver and spleen enlargement and fatty liver, with a liver CT value of 38.16 Hu and a spleen/liver CT intensity ratio of 0.63 (**Supplementary Figure 1**). The mean areas of abdominal subcutaneous and visceral fat were 28.57 cm^2^ and 28.78 cm^2^ respectively based on measurement of fat area by direct cross-sectional imaging using CT scan of the abdomen at the L1 vertebra level. In addition, abdominal ultrasound showed fatty liver, and the maximum diameter of the right lobe was 17.3 cm (RR: < 14 cm). Complications including polycystic ovary syndrome, atherosclerosis and pancreatitis were not observed.

**Figure 2:**
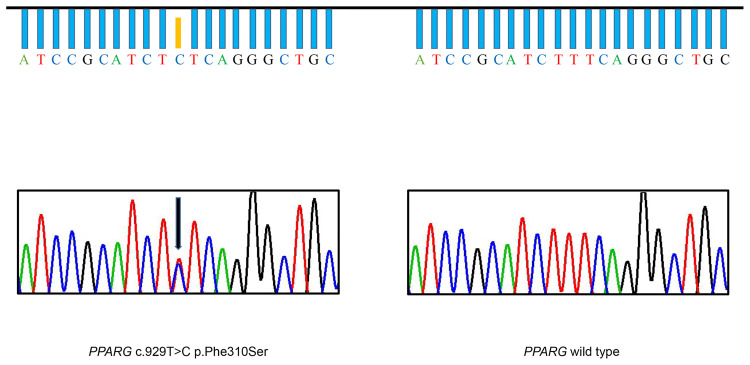
Identification of the F310S mutation. DNA sequencing chromatogram of a portion of the *PPARG* gene, illustrating that thymine was substituted with cytosine, leading to heterozygous substitution of phenylalanine by serine in the patient (arrow). The DNA sequencing chromatogram of the wild-type, lacking the mutation, is shown on the right.

Based on clinical manifestations, laboratory assessment, and imaging studies, FPLD syndrome should be considered. After obtaining the consent of the patient and her parents, we conducted a genetic analysis. Sequencing of genomic DNA showed that the patient had a novel, heterozygous mutation affecting the LBD of PPARγ c.929T > C (p.F310S), a variant that has not been previously reported (**Figure 2**). This mutation leads to the substitution of phenylalanine by a serine. According to PolyPhen-2 (Polymorphism Phenotyping v2) software (http://genetics.bwh.harvard.edu/pph2), this variant is predicted to have a potential impact on the stability and function of PPARγ protein. This mutation is predicted to be probably damaging with a score of 0.996 (sensitivity: 0.36; specificity: 0.97). The heterozygous mutation was not found in her brother (III-2) or parents (II-1, II-2). Similarly, they did not appear to have lipoatrophy or metabolic consequences. This pattern suggests that this mutation is a *de novo* mutation.

Taken together, the patient was ultimately diagnosed with FPLD3, T2DM, diabetic nephropathy, hypertriglyceridemia, liver dysfunction, fatty liver, hyperuricemia, and subclinical hypothyroidism. Ectopic fat deposition in nonadipose tissues may contribute to severe IR. Considering this situation, we decreased the insulin doses from 60 to 29 IU/day (insulin aspart injection 5 IU at breakfast, 5 IU at lunch and 5 IU at dinner, insulin degludec 14 IU at bedtime). In addition, she took oral hypoglycemic medication (combination of 30 mg/day pioglitazone and 1500 mg/day metformin), medication for protecting the liver (75 mg/day bicyclol), and medication for reducing proteinuria (5 mg/day benazepril). During hospitalization, fasting blood glucose and postprandial glycemia ranged between 9 - 10 mmol/L and 10 - 13 mmol/L, respectively. She was discharged to home with instructions for outpatient follow-up.

After 2 months of follow-up, she complained of asthenia and night sweats without hypoglycemia. In November of 2020, the insulin doses were increased from 29 to 54 IU/day (insulin aspart injection 10 IU at breakfast, 8 IU at lunch and 8 IU at dinner, insulin degludec 28 IU at bedtime) at a local hospital. Nevertheless, the symptoms did not improve. In January 2021, the patient returned to our hospital. Re-examination *via* abdominal CT revealed a significant improvement in the fatty liver, with a liver CT value of 49.42 Hu and a spleen/liver CT intensity ratio of 0.87 (**Supplementary Figure 1**). A repeated abdominal ultrasound showed an improvement in liver size compared with the preceding examination, with a maximum diameter of 14.5 cm in the right lobe. In addition, an increase in subcutaneous (34.79 cm^2^) and visceral fat (43.59 cm^2^) was shown in abdominal fat distribution evaluated by CT scan at the L1 vertebra level. The patient’s body weight increased from 42 to 45 kg during a follow-up period of 2 months. However, her triglyceride level rose to 7.90 mmol/L. Insulin treatment was discontinued and liraglutide 0.6 mg once daily was administered in addition to current treatment. However, the patient’s glycemic value was still inadequately controlled.

### *In Silico* Analysis of the F310S Mutation

**Figure 3:**
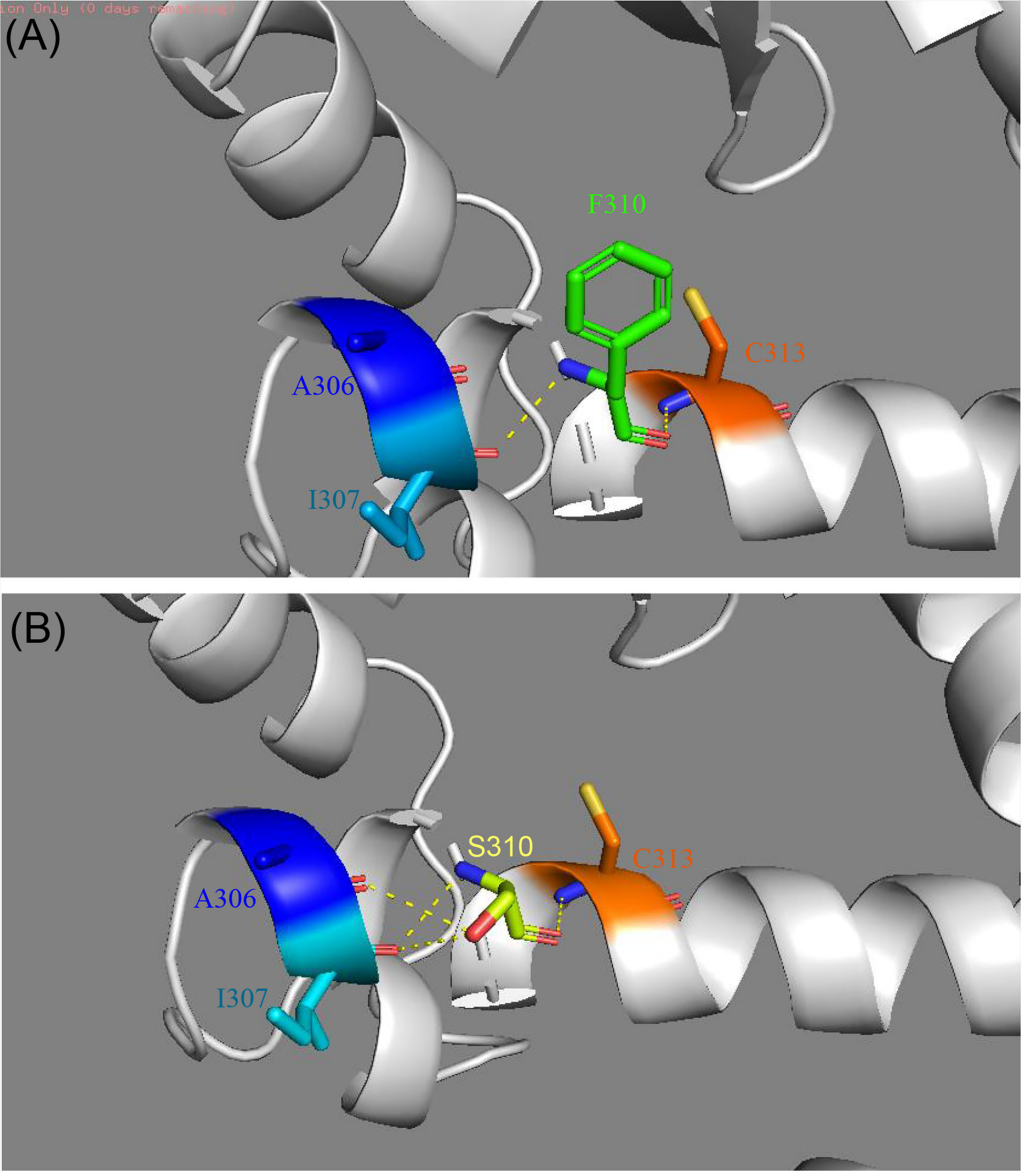
Comparison of the structure of modeled wild-type **(A)** and mutant PPARγ proteins **(B)** established by Swiss-Model. **(A)** The crystal structure of the wild-type PPARγ protein shows interactions between F310 and I307 and C313. **(B)** The crystal structure of the mutant PPARγ protein suggests that the F310S mutation alters the local hydrogen bond network of helix 3. Two new hydrogen bonds were formed after the F310S mutation, connecting to I307 and A306.

In the crystal structure of the PPARγ protein, Phe310, located in helix 3, participates in a hydrogen-bond network involving Ile307 and Cys313 (**Figure 3**). Mutations in this region could affect the ligand binding pocket, thereby interfering with ligand binding. To assess the consequences of the novel mutation F310S, the substitution of the amino acid phenylalanine at position 310 by the amino acid serine was simulated, and the optimal side-chain rotamer conformation was examined. The result showed that the mutant serine formed two new hydrogen bonds with alanine at position 306 and isoleucine at position 307. The mutation causes a change in the local hydrogen bond networks at this site. This change may affect the network of interactions needed to maintain the LBD in its active conformation.

### Clinical Findings in Patients with PPARG Mutations

**Figure 4:**
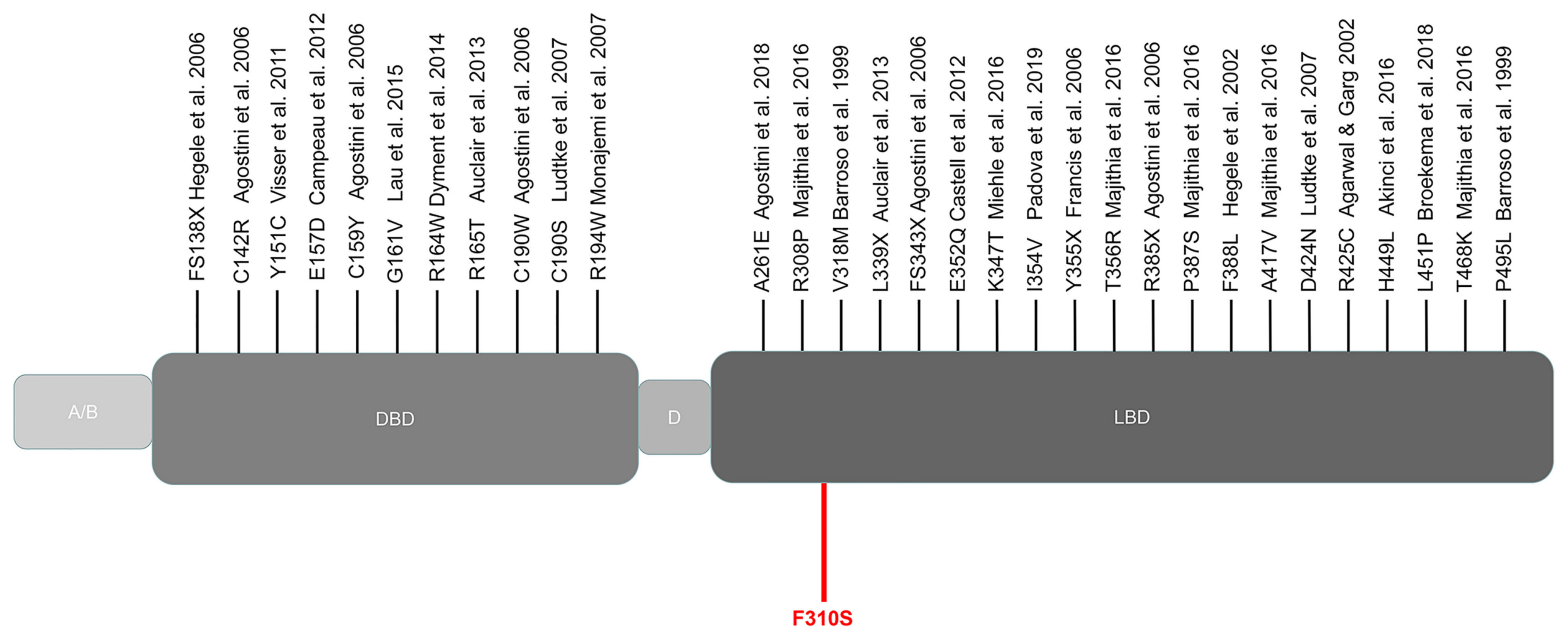
Schematic representation of the PPARγ domain structure and position of the mutations reported thus far from patients with FPLD3. Marked in red: novel mutation c.929T *>* C described in our study. A/B, N-terminal region; DBD, DNA-binding domain; D, hinge domain; LBD, ligand-binding domain.

We collected the location of the mutations thus far described for FPLD3 (**Figure 4**). Comparisons of clinical characteristics for the two groups are presented in **Table 1**. According to the inclusion criteria, 58 patients were eligible for the study. The patients’ general characteristics are summarized in **Table 1**. Overall, the number of patients was higher in the LBD group than in the DBD group. The prevalence of overweight was higher in the DBD group than in the LBD group (77% vs. 54%). For sex distribution, the majority of the patients were female in the two groups. Moreover, when divided by age (age *≤* 25 years), there were more patients in the LBD group than in the DBD group (29% vs. 11%), and all patients were female. This finding may suggest that females are more likely to develop FPLD3 than males. The first important feature of FPLD3 is the loss of adipose tissue. All patients had lipoatrophy at the limbs. The incidence of lipoatrophy in the gluteal region was also similar in the two groups (22% vs. 35%). Another important feature of FPLD3 is lipid accumulation. In the DBD group, accumulation was more likely to appear in the trunk or abdomen than in the face or neck (44% vs. 18%). Nevertheless, these results were similar in the LBD group (42% vs. 35%). In the DBD group, the prevalence rates of hypertension and cardiovascular disease were 77% and 14%, respectively, which were higher than those in the LBD group (58% and 9%), but there was no significant difference between the two groups. In addition, almost all patients presented with hypertriglyceridemia, with the exception of one patient in the DBD group. Other clinical characteristics of the two groups, such as DM, hepatic steatosis, hypercholesterolemia, acanthosis nigricans, hirsutism, and polycystic ovary syndrome, were not significantly different.

**Table 1:** Clinical data of FPLD3 patients from previous studies.

Characteristic	DBD (N = 27)	LBD (N = 31)	*P*-value
Female sex	18 (66)	24 (77)	0.36
Age ≤ 25 year	3 (11)	9 (29)	0.09
Overweight	21 (77)	17 (54)	0.06
Clinical lipoatrophy (gluteal region)	6 (22)	11 (35)	0.26
Clinical lipoatrophy (limbs)	27 (100)	31 (100)	1.00
Adipose tissue accumulation (face, neck)	5 (18)	11 (35)	0.14
Adipose tissue accumulation (trunk, abdomen)	12 (44)	13 (42)	0.84
Diabetes mellitus	19 (70)	24 (77)	0.54
Hypertension	21 (77)	18 (58)	0.11
Cardiovascular disease	4 (14)	3 (9)	0.69
Polycystic ovary syndrome	6 (33)^*^	11 (45)^*^	0.41
Hirsutism	8 (44)^*^	11 (45)^*^	0.92
Acanthosis nigricans	10 (37)	10 (32)	0.7
Hepatic steatosis	18 (66)	20 (64)	0.86
Hypertriglyceridemia	26 (96)	31 (100)	0.46
Hypercholesterolemia	7 (25)	7 (22)	0.76

Results are shown as n (%).

DBD, DNA-binding domain; LBD, ligand-binding domain.

Overweight: BMI ≥ 25kg/m^2^, Hypertriglyceridemia: triglycerides ≥ 1.84mmol/l, Hypercholesterolemia: total cholesterol ≥ 5.2 mmol/l or 240 mg/dl.

^*^All patients are female.

## Discussion

We report a case of FPLD3 in a Chinese female caused by a novel *de novo* mutation of *PPARG*. Subcutaneous fat was markedly diminished in her limbs, hips and face. She presented with severe metabolic disorders including IR, DM, hypertriglyceridemia, liver dysfunction and fatty liver. Moreover, the patient had an atrial septal defect and diabetic complications, which are rarely described in previous literature.

FPLD3 is a rare disease and was first reported by Barroso et al. in 1999 (8). In previous studies, the majority of patients were female, aged 8–71 years. The clinical phenotype of FPLD3 varies. Agarwal et al. described a white female with the D424N mutation who was diagnosed with hypertension at 12 years old (9). However, our patient’s blood pressure was continuously within the normal range. In addition, the patient with the FS138X mutation reported by Hegele et al., who was diagnosed with T2DM at a young age, was similar to our patient (10). Moreover, although the patient with the FS138X mutation was taking 100 U daily insulin and 1000 mg daily metformin, similar to our patient, she had insufficient glycemic control and presented with diabetic complications, including bilateral hearing impairment and peripheral neuropathy. Interestingly, except for our patient, facial lipoatrophy was rarely observed in patients with FPLD3, and only two patients were reported to have a loss of subcutaneous fat in the face in the literature (11, 12).

FPLD3 is a rare autosomal dominant genetic disorder. In our case, the patient carried a novel *PPARG* mutation c.929T > C. However, neither parent had this mutation. Therefore, this finding suggests that the variant is a *de novo* mutation. In previous studies, *de novo* mutations of other rare diseases have been reported occasionally (13, 14). However, such mutations have not been reported for FPLD3. Spontaneous mutations may have severe phenotypic consequences when they functionally affect relevant bases. In addition, when these mutations are carried in germ cells, they may be inherited.

Based on the results of prediction software, the F310S mutation was strongly predicted to be pathogenic. However, the biological effects of this mutation are still unknown. The R308P mutation, which is located near our F310S variant, was reported to be transcriptionally resistant to natural ligands, whereas its transcriptional function was nearly normal when tested with a synthetic ligand such as rosiglitazone (15). They found that the binding of pioglitazone can potentially alter the local protein conformation, thereby preserving transcriptional responsiveness to synthetic ligands. Correspondingly, the patient experienced a marked and sustained improvement in glycemic control and dyslipidemia following treatment with pioglitazone. However, different from the R308P mutation, although our patient received 30 mg/day pioglitazone for two months, she had poor glycemic control. This finding may suggest that the affinity of the F310S mutation to ligands may be different from that of the R308P mutation.

Structural modeling suggested that the phenylalanine to serine change at 310 would change the local hydrogen bond networks in helix 3, which may destabilize the ligand binding pocket. Thus, we hypothesized that a mutation in position 310 could result in reduced transcriptional activity. This hypothesis was partially supported. A previous study showed that PPARγ F310S mutation was associated with basal bladder tumors. Further biochemical and structure-function analysis showed that the F310S mutation decreases PPARγ activity through the destabilization of helix 12, thereby impairing the release of corepressors and the recruitment of coactivators (16). Moreover, mechanisms of negative dominance and haploinsufficiency have both been suggested to explain the pathogenicity of *PPARG* mutations. Miehle et al. suggested that similar changes in crystal structure might lead to similar changes in function (3). In previous studies, the V318M mutation also located in helix 3 has similar crystal structure changes (8). Functional studies confirmed that the V318M mutant exhibits transcriptional impairment and dominant-negative activity. In our case, the F310S mutation may have a similar effect.

Mutations, mostly located on DBD and LBD protein domains, have been reported to cause FPLD3. After reviewing the literature, no significant association was found between mutations located in different domains and clinical phenotypes. A possible explanation for this finding might be that the mutations located on the DBD or LBD could cause a similar phenotype through reduction of transcriptional activity in the mutant receptor, although the mechanisms are slightly different. In the DBD, the mutations disrupted the structure of PPARγ, thus affecting DNA binding, which results in a reduced ability to activate transcription (17). It has also been suggested that mutations could disturb the promoter release of PPARγ necessary for continued transcription (18). Correspondingly, in contrast to the wild type, mutation in the LBD could cause reduced transcriptional activity when interacting with ligand-mediated cofactors (such as rosiglitazone) (19). According to the latest study, a reduction of the abnormal protein’s transcriptional activity of ≥ 30% is sufficient to cause partial lipodystrophy (20).

PPARγ not only plays a critical role in adipogenesis, but also in cardiac development (21). In a study on mouse embryos, Barak et al. showed that PPARγ deficiency disturbs terminal differentiation of the trophoblast and placental vascularization, resulting in severe myocardial thinning and death (22). Subsequently, a series of studies have found that the expression of *PPARG* in mouse models is associated with cardiac hypertrophy, dilated cardiomyopathy, and the development of ventricular membranous septation (23–25). This cardiac hypoplasia was also observed in a patient with *PPARG* mutations. Our patient suffered from an atrial septal defect and was treated with surgical repair in 2006. In addition, two patients also suffered heart disease in their teenage years (15, 26). First, a 12-year-old girl with the Y355X mutation, was diagnosed with patent ductus arteriosus with pulmonary branch stenosis and an incomplete right bundle branch block at the age of 3 months. The second patient with the A261E mutation was diagnosed with dilated cardiomyopathy at age 20. The association between cardiac development and FPLD3 deserves further exploration.

In the patients reported thus far, we found that females accounted for the majority of patients with FPLD3. This gender difference has several explanations. First, the fat distribution is the difference between females and males. Premenopausal females accumulate adipose tissue in the gluteal region and subcutaneous tissue, whereas males tend to deposit fat in the abdomen (27, 28). Mutations in the *PPARG* gene could inhibit the differentiation of adipocytes, and lead to lipoatrophy. This finding implies that changes in appearance were more dramatic for females than males. Thus, the diagnosis of FPLD3 in females is much easier than in males. Second, estrogen receptor-β (ERβ) may play a role in FPLD3. ERβ, which belongs to the nuclear hormone receptor family, conveys the physiological signaling of estrogens. In 3T3-L1 preadipocytes, ERβ inhibited ligand-mediated PPARγ transcriptional activity and suppressed adipocyte differentiation (29). Similarly, ERβ knockout mice exhibited augmented PPARγ signaling in adipose tissue, which improved insulin sensitivity. A probable hypothesis is that interference of ERβ may further decrease PPARγ activity and cause clinical symptoms in a patient with *PPARG* mutations (30). Others have shown that women tend to have a greater expression of ERβ in adipose tissue than men (31). This finding might suggest that female patients are more prone to develop FPLD3 than male patients.

Regarding treatment for FPLD3, thiazolidinediones, which are high-affinity agonists of PPARγ leading to improved insulin sensitivity and adipogenesis, have been used (32). However, there is heterogeneity in individual responses to this treatment. In a study by Agostini et al., the proband was treated with 30mg/day pioglitazone, and exhibited clinical improvements (15). In another study, after 6 months of rosiglitazone treatment, the patient remained severely insulin resistant and showed little change in glycated hemoglobin levels (33). In terms of hyperglycemia management, metformin, a widely applied insulin sensitizer, is the first‐line hypoglycemic agent for the treatment of FPLD. The use of glucagon-like peptide-1 receptor agonists is associated with improvement of glycemic control and reduced insulin requirement. Moreover, in cases of severe IR, low-dose insulin was more effective than the large dose in improving IR and increasing insulin sensitivity (34). Cosmetic treatment was helpful for improving appearances and self-esteem in affected patients (35). In 2014, meterleptin, a recombinant analog of human leptin, was approved for patients with congenital or acquired generalized lipodystrophy in the US. However, the drug has not yet been approved for marketing in China.

Several limitations should be mentioned with this study. First, a functional study *in vitro* and *in vivo* could better reveal the biological effects of this mutation. We will conduct in-depth research on this topic in the future. Second, the leptin level was not evaluated.

## Conclusion

We reported a rare case of FPLD3 caused by a new *PPARG* mutation F310S. Moreover, we found no difference in clinical features between the DBD group and the LBD group. These results emphasize the importance of physical examination and genetic testing in young patients with severe metabolic syndrome in clinical practice.

## Data Availability Statement

The original contributions presented in the study are included in the article/**Supplementary Material**. Further inquiries can be directed to the corresponding author.

## Ethics Statement

This study was approved by the Ethics Committee of Tongji Hospital, Tongji Medical College, Huazhong University of Science and Technology, Wuhan, China. Written informed consent to participate in this study was provided by the participants’ legal guardian/next of kin. Written informed consent was obtained from the minor(s)’ legal guardian/next of kin for the publication of any potentially identifiable images or data included in this article.

## Author Contributions

All authors made a significant contribution to the work reported, whether that is in the conception, study design, execution, acquisition of data, analysis and interpretation, or in all these areas; took part in drafting, revising or critically reviewing the article; gave final approval of the version to be published; have agreed on the journal to which the article has been submitted; and agree to be accountable for all aspects of the work.

## Funding

This study was sponsored by the National Nature Science Foundation of China (Grant number 81700753). We gratefully acknowledge the patient and their families, as well as our colleagues for their cooperation and assistance throughout the course of this study.

## Conflict of Interest

The authors declare that the research was conducted in the absence of any commercial or financial relationships that could be construed as a potential conflict of interest.

## Publisher’s Note

All claims expressed in this article are solely those of the authors and do not necessarily represent those of their affiliated organizations, or those of the publisher, the editors and the reviewers. Any product that may be evaluated in this article, or claim that may be made by its manufacturer, is not guaranteed or endorsed by the publisher.
