# Supplementary material for: Case Report: A New Peroxisome Proliferator-Activated Receptor Gamma Mutation Causes Familial Partial Lipodystrophy Type 3 in a Chinese Patient
Source: Front Endocrinol (Lausanne). 2022 Mar 29;13:830708. doi: 10.3389/fendo.2022.830708 (PMC9001891; doi:10.3389/fendo.2022.830708)
Supplement: Supplementary file 1 [file Image_1.pdf]

Supplementary Material

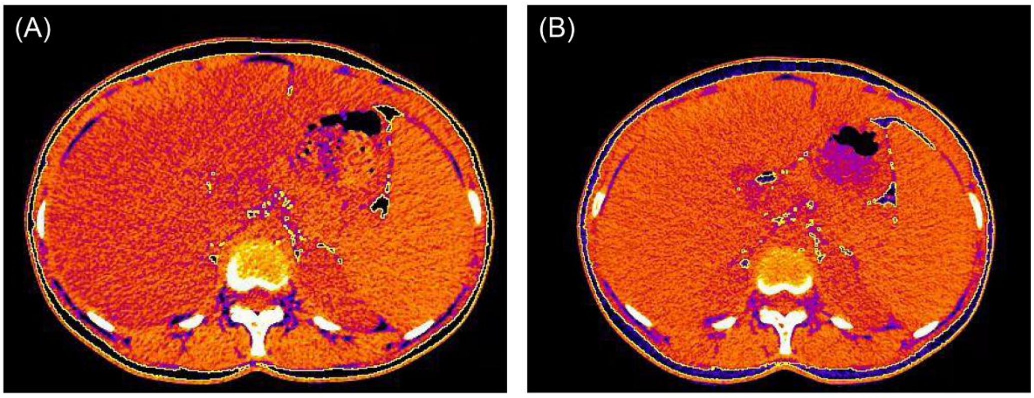

Supplementary Figure 1. TZD treatment improved the fatty liver in our patient. (A) Abdominal CT of the patient before treatment. Plain CT revealed liver and spleen enlargement and fatty liver, with a liver CT value of 38.16 Hu and a spleen/liver CT intensity ratio of 0.63. Abdominal fat distribution showed that the mean areas of abdominal subcutaneous and visceral fat were 28.57 cm^2 and 28.78 cm^2, respectively. (B) Re-examination via abdominal CT showed a significant improvement of the fatty liver, with a liver CT value of 49.42 Hu and a spleen/liver CT intensity ratio of 0.87. Abdominal fat distribution showed that the mean areas of abdominal subcutaneous and visceral fat were 34.79 cm^2 and 43.59 cm^2, respectively.
